# Anaesthetic Management of A Patient with Sturge-Weber Syndrome Undergoing Oophorectomy

**Published:** 2009-02

**Authors:** Manju Gandhi, Hemlata Iyer, Herman Sehmbi, Kavita Datir

**Affiliations:** 1 Professor, Department Of Anaesthesiology, BYL Nair Charitable Hospital & TN Medical College, Mumbai 400008; 2 Professor & Head of Department, Department Of Anaesthesiology, BYL Nair Charitable Hospital & TN Medical College, Mumbai 400008; 3 P.G. Student, Department Of Anaesthesiology, BYL Nair Charitable Hospital & TN Medical College, Mumbai 400008; 4 Lecturer, Department Of Anaesthesiology, BYL Nair Charitable Hospital & TN Medical College, Mumbai 400008

**Keywords:** Sturge-Weber Syndrome, Port Wine Angioma, Cerebro-Cortical Atrophy, Propofol

## Abstract

**Summary:**

The Sturge-Weber Syndrome (SWS) is a neurocutaneous disorder characterized by leptomeningeal and facial angiomas, neurologic and ocular manifestations. We report a case of oophorectomy for ovarian dermoid in a 14 year-old girl who was a diagnosed case of Sturge-Weber Syndrome. General anaesthesia was given for the procedure. The perioperative anaesthestic management is discussed in the present report.

## Introduction

Sturge^1^ (1879) first described the clinical features of the disease and Weber^2^ (1929) demonstrated the pattern of intracranial calcification and named it Encephalofacial Angiomatosis. The Sturge-Weber Syndrome (SWS) is a rare neurocutaneous syndrome of unknown etiology. It is primarily characterized by congenital cutaneous angiomas, neurological and ocular features.^3^

LeptomeningealAngiomasmay cause hemiparesis, hemianopsia, stroke like episodes,headaches, seizures, developmentaldelay, learning disabilities and mentalretardation.Vascularangiomasmay involve the nose, palate, gingiva, tongue, larynx and trachea posing challenge to laryngoscopy and intubation. Ocular features suchas glaucomamake smoothinduction and intubation necessary to limit the rise in intraocular and intracranialpressures. Perioperative hypoxemia, hypoglycemia, hypotension, ischaemiaand hyperthermia may precipitate seizures.The varied presentation and extent, that ranges fromlocalized superficial skin lesions to extensive systemic and airway involvement, makes the anaestheticmanagement challenging.^4^

## Case report

**Fig 1:**
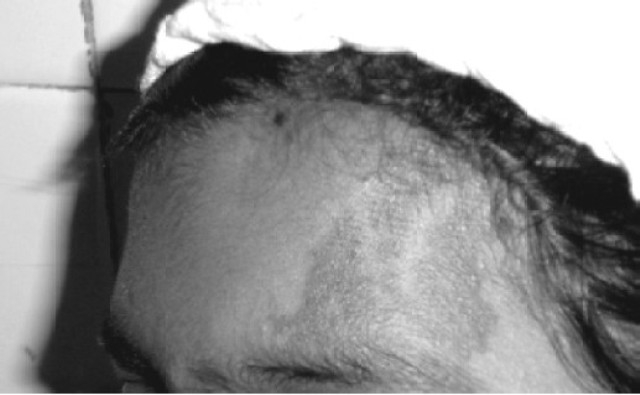
Left sided Port Wine Stain in V_1_ (first division of Trigeminal nerve) distribution (forehead)

**Fig 2:**
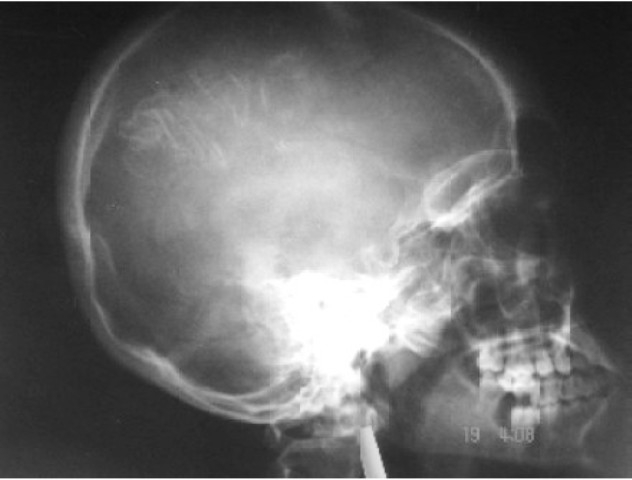
Lateral skull x-ray showing Tram Track calcifications in the left frontoparietal region

**Fig 3:**
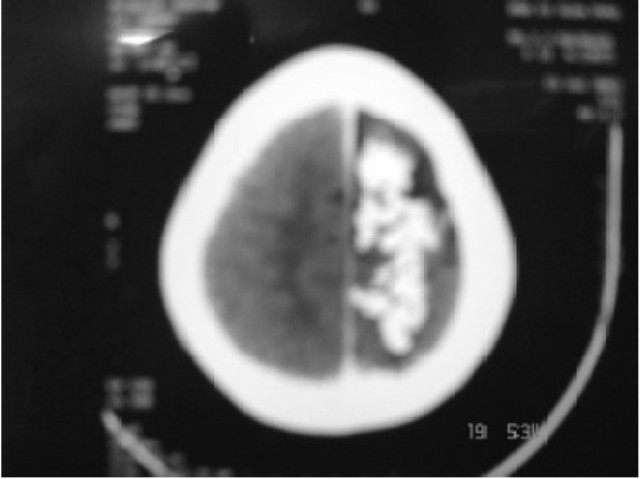
CT scan of brain showing left parietal angioma with dystrophic cerebral calcification

A 14-year-old girl (53 kg), a known case of SWS presented with a lump in lower abdomen since 4 years with progressive increase in size and discomfort. She had recurrent episodes of convulsions, focal with secondary generalization since 3 months of age with last episode 10 months ago and weakness in right upper extremity since childhood. The patient was on antiepileptic therapy (Carbamazepine 300 mg BD and Clobazam 10mg BD). Examination revealed a left sided 3 × 3 cm port wine stain in V_1_ distribution (first division of trigeminal nerve) over forehead, (Fig. 1). Right hemiparesis was also noted. There were no ocular features of SWS or any oropharyngeal hemangiomas on airway evaluation. Routine blood and urine investigations, ECG and chest X-ray were normal. Pelvic ultrasound revealed a 24 × 17 × 9 cm mass arising from the left ovary which was confirmed by CT scan. A lateral skull X-ray revealed gyriform calcifications (Tram-Track appearance) in the left front oparietal region, (Fig. 2). CT scan of brain revealed a left parietal angioma with dystrophic cerebral calcification, (Fig. 3). MRI scan of brain showed left parietal cerebral calcification, left cerebrocortical atrophy and signs of delayed myelination. Electroencephalography (EEG) showed attenuation of cerebral activity on the left side. A therapeutic drug level showed low normal levels of Carbamazepine 4 mcg/ml (normal 4-12 mcg/ml) and the dose was increased to 200 mg five times a day and the same dose was continued.

The patient underwent explorative laparotomy for oophorectomy. She was premedicated with i.v fentanyl 80 mcg, midazolam 1 mg, ranitidine 50 mg and i.v. ondansetron 4 mg 15 minutes prior to induction. Non invasive BP, ECG, SpO_2_ and EtCO_2_ were used to monitor the vitals. I.V. lidocaine 50mg was given to attenuate an intubation response. Preoxygenation was followed by induction with propofol 110 mg and vecuronium 5 mg. A careful gentle laryngoscopy and intubation were performed to minimize an intubation response. Anaesthesia was maintained with propofol infusion (100-150 mcg.kg^−1^.min^−1^), oxygen, nitrous oxide and vecuronium (three intermittent bolus doses of 1 mg each at 20 minute interval) with good haemodynamic stability. Fentanyl 50 mcg was repeated intraoperatively. At the end of surgery, the neuromuscular blockade was reversed with neostigmine 2.5 mg and atropine 1.2 mg. Extubation response was attenuated using lidocaine 50 mg. The patient was shifted to the post anaesthetic care unit (PACU) and post-operaive pain relief was provided with i.v. tramadol 50 mg TDS. The recovery was uneventful and the patient was discharged from the hospital on the 10^th^ postoperative day.

## Discussion

The SWS patients may present for facial and cosmetic surgeries, dental procedures, trabeculectomy, goniotomy, examination under anaesthesia, seizure control surgery or other surgeries.^4^–^6^

Angiomas may involve the airway (nose, palate, gingiva, tongue, larynx and trachea) leading to difficult mask ventilation, laryngoscopy and intubation, apart from bleeding caused by rupture. Intubation should be done using a soft, lubricated, non styleted, cuffed endotracheal tube by expert anaesthesiologist. Because these vessels have abnormal autoregulation, the intraoperative blood pressure should be well controlled.^7^ Emotional agitation may increase the volume of haemangiomas markedly and may necessitate avoiding surgery in the morning.^5^ Vascular changes may also affect the dura, brain, pituitary, lungs, spleen, and other organs.^4^ A thorough evaluation ruled out such lesions in our patient. Cutaneous features include Port Wine Stain, facial or extrafacial, truncal or on the extremities. Its presence in the V_1_ distribution (first division of the trigeminal nerve) is associated with a higher risk of epilepsy or glaucoma.^3^ Ocular features include glaucoma (30-71%) most commonly, bupthalmos, choroidal hemangioma, strabismus and others.^3^ They may require avoidance of drugs that can cause an increase in intra ocular pressure (IOP) such as succinylcholine and ketamine. Anticholinergics should be avoided in patients with narrow angle glaucoma.

Cerebral angioma undergoing vascular steal may lead to cerebral ischaemia resulting in intractable and recurrent seizures, status epilepticus, and recurrent vascular events. Seizures are the most common neurologic manifestations, mostly focal, though may be secondarily generalized. Onset of seizures before the age of 2 years suggests a greater chance of refractoriness, mental retardation and neurologic involvement. Early onset of seizures, extensive angiomas, progressive neurologic involvement, hemiparesis and deteriorating cognitive functions are predictors of poor outcome^3^. Our patient had an early onset (3 months of age), focal with secondarily genaralised convulsions, with a recurrent course, but they were well controlled with carbamazepine and clobazam. Preoperatively, antiepileptic therapeutic drug level was done to guide the therapy. Since the therapeutic level of carbamazepine was low, its dose was increased from 600 mg/day to 1000 mg/day. Concommitant chronic use of antiepileptics can affect metabolism of some anaesthetic agents. Low cognitive understanding and mental retardation can interfere with doctor-patient communication perioperatively causing emotional stress, increased blood pressure and consequent swelling of angiomas. It is advisable to interview the patient preoperatively and establish a good rapport. Succinylcholine is avoided in hemiplegia due to its effect on serum potassium^7^,^8^. We used a fentanyl-propofol-vecuronium combination to achieve and maintain haemodynamic stability intraoperatively^5^. Light plane of anaesthesia, bucking, straining and airway obstruction during induction or emergence can cause an increase in intraocular and intracranial pressures and should be prevented^9^. Events like hypoxemia, hypoglycemia, hypotension, ischaemia and hyperthermia may precipitate status epilepticus and should be avoided^3^.

In case of dental procedures with oral angiomas, hemostasis may be difficult to achieve and an overzealous approach should be avoided. Yamashiro and Furara^5^ reported cancellation of gingivectomy due to potential for excessive bleeding. The presence of pre-existing neurodeficit (right sided hemiparesis) precluded the use of epidural anaesthesia in our patient. Despite the claims of proconvulsant actions of propofol, there is significant evidence to the contrary. Propofol has been successfully used to treat status epilepticus^10^ and drug-induced seizures^11^. Yamashiro and Furura^5^ successfully used propofol in a patient of SWS for oral surgery. Cochran et al^12^ reported that it is advisable to avoid propofolin patients at risk for seizures and poorly controlled epilepsy. Our patient was well controlled with anticonvulsants, hence we proceeded with the use of propofol. We encountered no problem or complications which can be attributed to careful screening, monitoring and anaesthetic management.

A good knowledge and an understanding of SWS, careful preoperative evaluation and a proper anaesthetic management plan are of utmost significance. Achieving control of convulsive disorder perioperatively, attenuation of haemodynamic responses during airway manipulation and surgery, careful intubation/extubation, avoiding trauma to angiomatous lesions, limiting the rise in intra-ocular and intracranial pressures, with adequate pain relief leads to safe anaesthetic management of these patients.
